# Stringent Primer Termination by an Archaeo-Eukaryotic DNA Primase

**DOI:** 10.3389/fmicb.2021.652928

**Published:** 2021-04-13

**Authors:** Jan Bergsch, Jean-Christophe Devillier, Gunnar Jeschke, Georg Lipps

**Affiliations:** ^1^Institute of Chemistry and Bioanalytics, University of Applied Sciences Northwestern Switzerland, Muttenz, Switzerland; ^2^Department of Biology, Institute of Biochemistry, ETH Zurich, Zurich, Switzerland; ^3^Department of Chemistry and Applied Biosciences, ETH Zurich, Zurich, Switzerland

**Keywords:** replication, priming, archaea, genome maintenance, regulation, DNA protein interaction

## Abstract

Priming of single stranded templates is essential for DNA replication. In recent years, significant progress was made in understanding how DNA primase fulfils this fundamental function, particularly with regard to the initiation. Equally intriguing is the unique property of archeao-eukaryotic primases to terminate primer formation at a well-defined unit length. The apparent ability to “count” the number of bases incorporated prior to primer release is not well understood, different mechanisms having been proposed for different species. We report a mechanistic investigation of primer termination by the pRN1 primase from *Sulfolobus islandicus*. Using an HPLC-based assay we determined structural features of the primer 5′-end that are required for consistent termination. Mutations within the unstructured linker connecting the catalytic domain to the template binding domain allowed us to assess the effect of altered linker length and flexibility on primer termination.

## Introduction

During cellular DNA replication, the inability of replicative polymerases to initiate synthesis on a single strand requires the activity of DNA primase. Its function is the *de novo* synthesis of oligonucleotides on single stranded DNA serving as a substrate for a DNA polymerase that in turn extends the primer at its 3′-OH terminus. As a consequence of the unidirectional nature of DNA synthesis, a new primer must be formed for every Okazaki fragment on the lagging strand (Kornberg, 1980). Since primases are more error prone than the replicative polymerases (Sheaff and Kuchta, 1994) the primers have to be replaced after replication by a proofreading enzyme (Hombauer et al., 2011; Liberti et al., 2013). By using RNA, the replication machinery creates a marker for proofreading of primase-generated stretches during Okazaki fragment processing. Archaeal primases that form chimeric or DNA primers (Beck and Lipps, 2007) thus need to terminate reliably at short primer lengths to prevent errors from affecting genome stability.

Primer synthesis can be divided into two main phases, the first phase being the synthesis of the initial dinucleotide while the second phase is the repeated elongation of the dinucleotide/template (Kuchta and Stengel, 2010). Typical DNA primases synthesize primers of a defined length, usually between four and 20 nucleotides. This is in contrast to DNA polymerases whose product length is not intrinsically limited and which even use sliding clamps to increase the processivity (Mizrahi et al., 1985). Primases produce a narrow length distribution of primers. Consequently, a mechanism must be in place to terminate the repeated elongation of the primer-template at certain primer lengths. Interestingly, the termination of primer synthesis at a defined length is not brought about by a signal from another component of the replisome. Primer termination is readily observed in primase assays in the absence of further replicative enzymes. Evidently, the mechanism that limits primers to a defined length is an inherent property of the primase itself.

Two distinct classes of primase enzymes exist, viz. the bacterial and bacteriophage DnaG type primases and the archeoeukaryotic primases (AEP) (Iyer et al., 2005). Both types differ in sequence and structure but carry out the same crucial function in replication (Bergsch et al., 2019).

In the eukaryotic primosome, primase forms a complex with DNA polymerase α (Polα), yet primer termination at a specific length is observed in assays without Polα. For instance, the primers formed by *Drosophila melanogaster* DNA primase have a typical length of 15 nt (Conaway and Lehman, 1982), whereas human primase forms a primer with a length of nine nucleotides (Baranovskiy et al., 2016a). The specific typical length of primers characteristic of a primase enzyme has been termed a unit length primer (Desogus et al., 1999; Baranovskiy et al., 2016a).

Stability and amenability to structural investigation have contributed to the role of archaeal primases as model enzymes of the AEP superfamily (Keck and Berger, 2001). The primase of the archaeon *Methanococcus jannaschii* produces primers of a unit length of 7 nt, but can extend them to form products of twice this length or a distribution of longer products (in the manner of a polymerase) depending on the pH (Desogus et al., 1999), pointing toward a role of the protonation of one or multiple residues in the regulation of primer length determination. The crenarchaeon *Saccharolobus solfataricus* primase generates primers with a length distribution from 14 to 18 nt (Yan et al., 2018).

**FIGURE 1:**
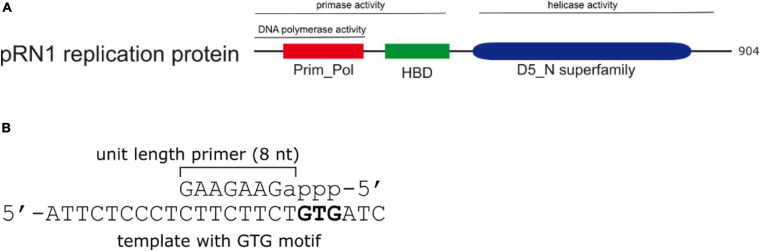
Domain structure and primer product of the pRN1 replication protein. **(A)** Domain structure and activities of the pRN1 replication enzyme. The pRN1 primase consists of an N-terminal catalytic domain (Prim_Pol, cd04859 amino acids 40–248, red), a flexible linker (amino acids 249–260) and a C-terminal helix bundle domain (HBD, pfam13010 amino acids 256–370, green). The full-length replication protein also contains a C-terminal helicase domain (blue) which is independent to the primase domain (Beck and Lipps, 2007). This study was performed with deletion mutants comprising the Prim_Pol, linker, and HBD. **(B)** The enzyme synthesizes an 8 nt long mixed RNA/DNA primer. Ribonucleotides are in lower case, deoxyribonucleotides in capitals. The primase requires a GTG recognition motif in the template, downstream of which the primer is formed.

We have been investigating the primase enzyme from the plasmid pRN1 in *Saccharolobus islandicus* (pRN1 primase). Homologs of this plasmidal replication enzyme are present in related plasmids from the pRN plasmid family (Peng et al., 2000). This enzyme is a particularly compact, yet highly active and stable primase, allowing for detailed investigation of the primase mechanism by quantitative assays as well as structural approaches (Figure 1).

The primase activity of the replication protein ORF904 from the archaeal plasmid pRN1 resides in the N-terminal half of the enzyme more specifically, the catalytic Prim_Pol domain (aa 40–248; compare Figure 1) and the helix-bundle domain (HBD, aa 256–370) are required for activity (Beck et al., 2010). This enzyme produces chimeric primers with a unit length of 8 nt, of which the first NTP incorporated (the 5′-terminal of the primer) is constitutively a ribonucleotide, while the remaining part of the primer is DNA (Beck and Lipps, 2007).

Although the catalytic domain alone is sufficient to support DNA polymerase activity, the HBD is indispensable for primase activity. The exact contribution of the HBD toward primer synthesis has long remained unclear. Recently, we could show that the HBD is able to bind sequence-specifically the template and two nucleoside triphosphates (Boudet et al., 2019). We therefore suggest that the HBD prepares the initial dinucleotide synthesis by binding and preassembling the three substrates (template, initiating ribonucleotide and elongating deoxyribonucleotide) required for the reaction to occur. Such a contribution to primer synthesis would explain why the HBD is required for *de novo* synthesis of a primer but not for elongating a primer hybridized to a template. The pRN1 primase enzyme is a single subunit primase in contrast to most eukaryotic primases which are heterodimeric. Noteworthy, primer synthesis is only observed when catalytic domain and HBD are covalently coupled. A mixture of the isolated domains is not enzymatically active, underscoring the importance of the linker region between catalytic domain and HBD (Beck et al., 2010). In the crystal structure of the primase, residues K250 to F260 situated between the catalytic domain and the HBD are flexible (Beck et al., 2010).

The DNA binding interface of the HBD resembles the DNA binding interface of the primase large subunit (PriL) of cellular archaeo-eukaryotic primase (Boudet et al., 2019). The contribution of PriL for primer synthesis is not very well defined on a molecular level either, but increasing evidence suggests that the helical p58C subunit of PriL binds the template and might also bind the initiating nucleotide (Baranovskiy et al., 2016a, b). Thus, PriL and HBD appear to share a similar functional role in primer synthesis by preparing the dinucleotide synthesis. Moreover, human p58C is able to specifically bind the phosphorylated 5′ primer end hybridized to the template, suggesting that the primer/template remains bound to the PriL during the primer synthesis catalyzed by the active site of the catalytic subunit PriS (Baranovskiy et al., 2016a).

Thus, for the archaeo-eukaryotic primases the following mechanistic picture emerges: These primases have two functionally conserved domains, the catalytic domain carrying out the enzymatic reaction of nucleotide condensation and a helical domain responsible for preparing primase initiation, or more specifically, preparing dinucleotide formation (Boudet et al., 2019). While there is meanwhile a rather good understanding of how primer synthesis is initiated, the mechanism of primer termination at specific primer lengths is not well investigated and understood. The following hypotheses explaining termination of primers specifically at unit length have been envisaged:

Firstly, termination of primers at the observed length might be a stochastic effect based on the low processivity of primases (Spiering et al., 2017). This would inevitably lead to a broad distribution of primer lengths.

Secondly, the occurrence of primer length “counting” can be explained by taking into consideration that primase remains bound to the 5′-end of the nascent primer during catalysis of the primer elongating reaction while the 3′-end of the primer is contacted by the active site. The dual interaction allows the enzyme to measure the linear distance between these sites. Termination occurs as soon as a primer length is attained that corresponds to the maximum distance attainable by the malleability resulting from flexible parts of the structure. In fact, specific interaction of the 5′ end with the PriX subunit of *S. solfataricus* primase lead Yan et al. (2018) to propose a molecular “caliper model” to explain the termination of primer extension in the *S. solfataricus* primase.

Also based on concomitant binding of both primer ends, a third mechanism of primer termination has been proposed. This model takes into account that formation of the primer-template duplex results in a change from linear single stranded DNA to a double strand, which tends to adopt a helical conformation. Consequently, the nascent helix forces the enzyme domains at the ends of the primer to rotate relative to each other around the axis of the template DNA. This mode of termination has been proposed for the human primosome based on structural evidence of sterical hindrance between p58_*N*_ and p58_*C*_ leading to a clash exactly after 9 nt (Baranovskiy et al., 2016a).

The pRN1 primase forms a unit-length primer of eight nucleotides (Lipps et al., 2003). In order to better understand the elongation cycle of the pRN1 primase and its specific termination we first investigated in more detail the substrate requirements of the primase initiation and primase extension activity, building on recent structural insights. Next, we investigated which of the proposed mechanisms of primer termination could apply to pRN1 primase. To this end, we took advantage of the covalent coupling of the catalytic domain and the HBD which allowed us to rigorously test the mechanistic requirements of primer termination by constructing mutants with linker sequences differing in length and flexibility. Finally we built a structure model of pRN1 primase in complex with the substrate DNA.

## Materials and Methods

### Recombinant Expression and Purification of Proteins

The pRN1 primase and mutants thereof (amino acids 40–370) was expressed recombinantly in *Escherichia coli* BL21 (DE3) using the expression plasmid pET28c(+) and a c-terminal His_6_-tag. Point mutants were generated according to the QuickChange PCR protocol involving two mutagenic oligonucleotide primers (synthetic primers were obtained from Microsynth AG) and Pfu polymerase, parental DNA was digested with *Dpn*I. The incorportion of mutations was verified by Sanger sequencing (Microsynth AG).

Cultures were grown in LB medium (Miller) up to OD_600 nm_ = 0.6, then induced by addition of 1 mM IPTG and fermentation was continued for 16 h at 30°C. Cells were collected by centrifugation at 8,500 *g* for 20 min and the pellet resuspended in 20 mL of lysis buffer (50 mM Na_2_HPO_4_, 100 mM NaCl, 10 mM MgCl_2_ pH 8.0) per 2 L culture to which 0.01% v/v Triton X-100 was added prior to sonication. The whole cell extract was cleared by centrifugation at 34,000 *g* for 20 min. The supernatant was loaded onto a Co^2+^ Talon affinity chromatography column (5 mL), eluted with an imidazole gradient. Fractions containing the target proteins (∼ 15 mL) were pooled and dialyzed against storage buffer (50 mM HEPES, 100 mM NaCl, 10 mM MgCl_2_ pH 7.0). The resulting solution was concentrated by ultrafiltration (10 kDa MWCO) to approximately 2 mL and further purified by size exclusion chromatography on a 60 cm × 16 mm Superdex 75 column (GE healthcare) using storage buffer (50 mM HEPES, 100 mM NaCl, 10 mM MgCl_2_ pH 7.0) as the mobile phase. Peak fractions were pooled and concentrated by ultrafiltration to approximately 0.5 mM. Protein concentrations were measured using the theoretical extinction coefficient at 280 nm. The preparation typically yielded between 20 and 50 mg protein per liter of culture volume.

### Primase Assay

Reactions were performed in 15 mL volume containing 1× NEB-1 buffer (New-England Biolabs; 10 mM Bis-Tris-Propane-HCl, 10 mM MgCl2, 1 mM DTT, pH 7.0), 50 μM of the relevant NTPs, 5 μM ssDNA template, and 0.25 μM of enzyme. Synthetic DNA oligonucleotides used as templates or primers were obtained from Microsynth AG, and were of HPLC-purified quality. Some assays used oligonucleotides produced by pRN1 primase previously isolated by preparative HPLC. If a primer-template duplex DNA was used as a substrate for the primase essay, the strands were annealed by the following temperature program without further purification: 98°C for 2 min; 70°C for 5 min; 50°C for 10 min; 40°C for 5 min; 25°C for 2 min (in reaction buffer).

The assays were incubated at 55°C for 20–120 min, then stopped by addition of 15 mL 15 mM EDTA pH 8.0 and incubated on ice for 5 min. The resulting mixtures were centrifuged 5 min at 16,000 *g* prior to analysis by HPLC.

### Preparative and Analytical HPLC, MS

The products of primase reactions were separated and analyzed on an XBridge C18 3.5 mm (3.0 mm inner diameter 150 mm length) HPLC column (Waters) at 55°C using an Agilent 1100 Series HPLC system. For analytical purposes 20 μL of sample were injected. A gradient elution was carried out using as solvent A 8 mM triethylamine and 200 mM hexafluoroisopropanol in water and as solvent B a mixture of 50% v/v A and 50% v/v methanol. The mobile phase was delivered at a flow rate of 1 mL/min, with a gradient from 5% B to 50% B over 8 min. Analytes were detected by measuring absorbance at 260 nm using a diode array detector (Gilar et al., 2002). Peak areas in HPLC chromatograms were integrated using the program PeakFit v4.12. The amounts of primase products were calculated from the peak areas based on the extinction coefficients of the different oligonucleotides according to the conversion factors given in Supplementary Table 1. Rates of nucleotide incorporation and primer synthesis were calculated in units of mol dNTPs or mol primers per mol enzyme per minute.

For preparative HPLC the injection volumes was increased to 50 or 80 μL and the gradient was extended to 20 min. Peak fractions were manually collected and lyophilized using a Christ Alpha 2-4LD freeze dryer. The identity and purity of products were verified by analytical HPLC and by MALDI-TOF mass spectrometry. Samples for MALDI-TOF analysis were dissolved in water and spotted onto a 2′,4′,6′-Trihydroxyacetophenone (THAP) matrix and were analyzed at the mass spectrometry facility of the University of Bern.

### Structure Modeling

For modeling the link between the DNA sections GCAG, bound to the Prim_Pol domain and GTGCTCA, bound to the HBD, we used the backbone pseudo-rotamer library for RNA developed by Humphris-Narayanan and Pyle (2012). The library at 5° resolution (Pyle Lab, 2021) contains 577 backbone conformations for each nucleotide. We first determined which of these conformations superimposed with the given coordinates of the two terminal G nucleotides with a root mean square deviation (RMSD) below a threshold value Δ_RMSD_. For all link combinations we predicted the coordinates of the P and C4′ atoms of the first nucleotide of the second DNA section by superimposing the pseudo-rotamer from the library with the coordinates of the last nucleotide of the first DNA section. Likewise, we predicted the coordinates of the C4′ atom of the last nucleotide of the first DNA section by superimposing the pseudo-rotamer with the coordinates of the first nucleotide of the second DNA section. This provided two triplets of C4′ (*i*)-P(*i* + 1)-C4′ (*i* + 1) coordinates that we superimposed. Link geometries were accepted at this stage if the superposition RMSD was again below Δ_RMSD_. From these link geometries we selected those for which the nucleobases did not clash, obtaining 9,046 link geometries at Δ_RMSD_ = 0.25 Å and 53,466 link geometries at Δ_RMSD_ = 0.5 Å. We then removed all link geometries with clashes between the two domains as well as those with a CA-CA distance longer than 49.4 Å between the C-terminal residue of the CAD and the N-terminal residue of the HBD, which are joined by a 12-residue linker (G249-F260). This reduced the number of possible link geometries to 2 at Δ_RMSD_ = 0.25 Å and to 27 at Δ_RMSD_ = 0.5 Å. In both ensembles, the peptide linkers were then constructed by YASARA (Krieger et al., 2009), followed by geometry optimization in YASARA. The two primase conformers at Δ_RMSD_ = 0.25 Å constitute the two alternative models presented. The broader ensemble contained conformations resembling the two models except for relative domain rotations by a few degrees and was therefore not considered further.

## Results and Discussion

### Depending Upon Substrate pRN1 Primase Works as a Primase or as a DNA Polymerase

In contrast to other archaeo-eukaryotic primases, pRN1 primase strictly uses deoxynucleotides for primer elongation, whilst the first nucleotide of the primer is a ribonucleotide (Figure 1). In addition to its primase activity the pRN1 primase also has DNA polymerase activity. Using circular single-stranded DNA template extension products of several kilobases are observed (Lipps et al., 2003). However, in contrast to the first observation, when a single-stranded template containing the 5′-GTG-3′ motif is offered as substrate, a primer of the length of eight nucleotides is the major product (Beck and Lipps, 2007). It thus appears that the pRN1 primase operates in two distinct modes, the “primase mode” and the “DNA polymerase mode.” We therefore analyzed the products of the pRN1 primase reaction assays *via* HPLC chromatography using different defined short templates and primer/templates in order to understand how pRN1 primase selects its “operating” mode.

When pRN1 primase is supplied with a GTG containing template (Table 1, substrate GTG) a primer of a length of eight nucleotides is synthesized when ATP, dATP and dTTP are included in the reaction mix. A single base change in the GTG recognition motif (substrate CTG) abrogates primer synthesis in line with previous experiments on the template specificity of the primase activity (Beck and Lipps, 2007) (chromatograms shown in Supplementary Figure 1).

**TABLE 1:** DNA substrates used in this study.

**Name**	**Sequence**	**Description**	**Reaction product**
GTG	5′-AAAAAAATTTTTT**GTG**ATC	GTG containing template	Unit length primer
CTG	5′-AAAAAAATTTTTTC**TG**ATC	Non-GTG containing template	No reaction
A_6_/GTG	AAAAAA-5′ 5′-AAAAAAATTTTTT**GTG**ATC	Synthetic primer/template	Run-off product
pppaA_5_/GTG	AAAAAappp-5′ 5′-AAAAAAATTTTTT**GTG**ATC	Genuine primer/GTG template	Elongation to unit-length primer
pppaA_5_/CTG	AAAAAappp-5′ 5′-AAAAAAATTTTTTC**TG**ATC	Genuine primer/non-GTG template	Run-off product
T_8_GTGT_3_	5′-TTTTTTTT**GTG**TTT	Standard primase substrate	Unit length primer
Mixed	5′-ATTCTCCCTCTTCTTCT**GTG**CACTCTTCTTCTCCC	Mixed template for primer length determination	Unit length primer

*Shown are the sequences of the different primers and templates with the GTG motif in the template in bold. Deoxynucleotides are in upper-case letters, the triphosphorylated adenosine at the 5′ end of the genuine primer is indicated as appp. For each substrate a short description as well as type of products found by HPLC are given.*

When incubating pRN1 primase with substrate GTG (A_7_T_6_**GTG**ATC) in the absence of dTTP an only six base long primer (pppaA_5_) is synthesized. Primer synthesis is aborted at this length due to the lack of the cognate nucleotide dTTP (see Supplementary Figure 1 trace B).

Unlike a synthetic DNA primer, such a primer formed by pRN1 primase contains a triphosphorylated ribo-adenosine at the 5′ end (primer substrate pppaA5, Table 1). We isolated ∼ 10 nmol of this primer to serve as substrate for subsequent assays by a preparative HPLC purification and confirmed the identity of the aborted primer product by MALDI mass spectrometry (Supplementary Figure 2).

Next, we tested whether pRN1 primase can restart primer synthesis from a preformed primer/template. For this purpose we hybridized template GTG with the abortion product pppaA_5_ and alternatively with chemically synthesized oligodeoxynucleotide (Table 1, primer A_6_). Both primers have the same base sequence but differ at the 5′ terminal backbone sugar and by the 5′ triphosphate group, which is only present in the enzymatically synthesized abortion product.

**FIGURE 2:**
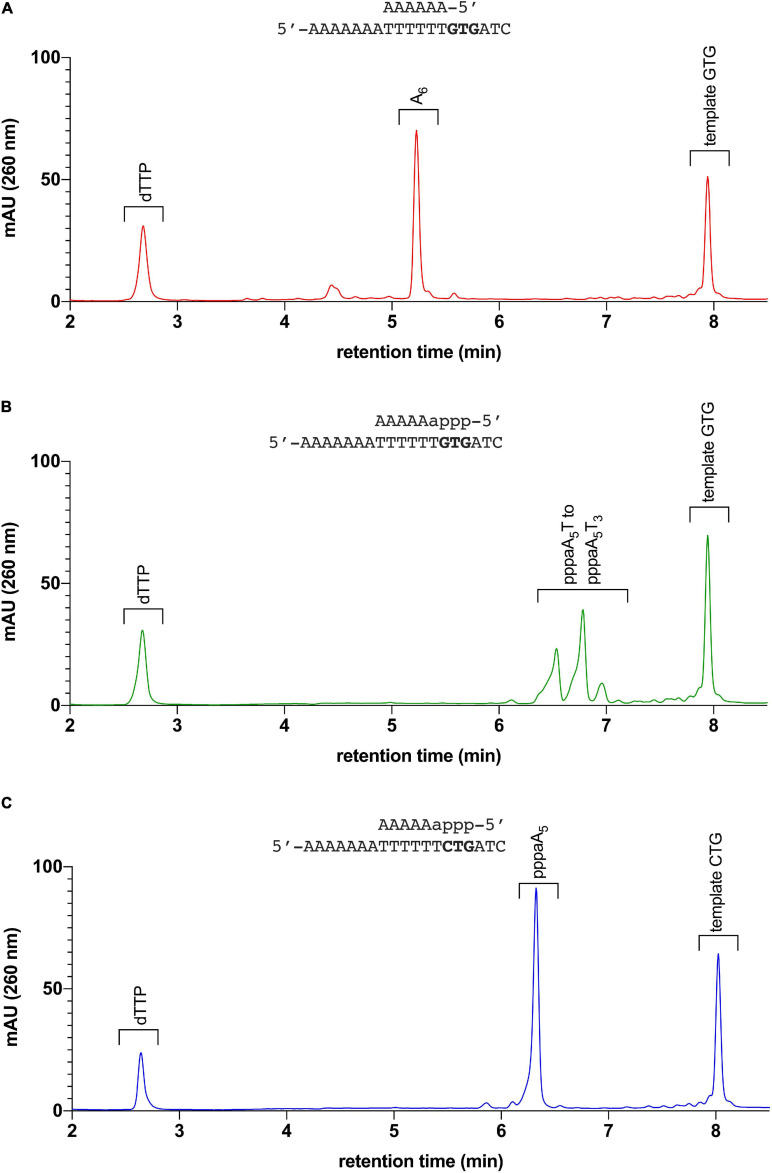
Elongation assay: pRN1 enzyme incubated with different primer-template substrates. **(A)** Primer-template combination A6/GTG: The synthetic 6 nt primer is not elongated by the primase in presence of dTTP. **(B)** Primer-template combination pppaA5/GTG (6 nt primer with triphosphorylated ribonucleoside at 5′ terminus): The 6 nt primer is elongated by the primase in presence of dTTP. Incorporation of dTTP by the primase leads to formation of unit length products (primase mode, for comparison of primers formed with this template, refer to Supplementary Figure 1). **(C)** Primer-template combination pppaA5/CTG: The primer pppaA5 on a template lacking the recognition sequence GTG is not elongated. No unit length products are observed with template CTG instead of GTG.

The chemically synthesized DNA primer/template (substrate A_6_/GTG) is not extended by the pRN1 primase to form unit length primers; only slow formation of the run-off product through its DNA polymerase activity occurs, albeit at a much lower rate compared to primase activity (see below in Table 2 for the incorporation rates). Thus, an extension product of the primer unit length of eight nucleotides, as expected for a primase reaction, is not observed in the HPLC chromatogram of the assay containing a synthetic DNA primer (Figure 2A).

**TABLE 2:** Nucleotide incorporation rates of the pRN1 primase with different substrates.

**Substrate**	**Rate [min^–1^]**	**Enzyme**
***dNTP incorporation rates in “primase mode”***		
GTG	2.849 ± 0.086	Primase (AA40–370)
pppaA_5_/GTG	1.341 ± 0.107	Primase (AA40–370)
pppaA_5_/CTG	<0.1 (no product detected by HPLC)	Primase (AA40–370)
***dNTP incorporation rates in “DNA polymerase mode”***		
A_6_/GTG	0.076 ± 0.003	Primase (AA40–370)
A_6_/GTG	0.107 ± 0.009	Primase w/o HBD (AA40–250)
A_6_/CTG	0.049 ± 0.011	Primase (AA40–370)
A_6_/CTG	0.223 ± 0.036	Primase w/o HBD (AA40–250)

*Assays were performed as described in section “Materials and Methods,” with 20 min incubation for “primase mode” measurements (also refer to Figure 2) and 120 min incubation for “DNA polymerase mode” measurements. The reaction products were quantified by HPLC. The values reported are the average of five replicates and errors range estimated as standard deviation.*

In contrast, with the enzymatically produced primer hybridized to the same template (substrate pppaA_5_/GTG) the primer is extended to the unit length primer but not to the run-off extension product (Figure 2B). We refer to this activity of pRN1 enzyme, leading to a unit-length extension product shorter than the available template as the “primase mode.”

To further corroborate this finding we repeated the assay with the substrate pppaA_5_/CTG. This substrate has a single point mutation in the GTG recognition motif, which is required by pRN1 primase for *de novo* primer synthesis. With substrate pppaA_5_/CTG, an eight nucleotide long primer is not observed (Figure 2C) in line with the observation that initiation of primase mode requires the GTG motif in the template strand. While the 30 min incubation time of the primase assay used here results in no detectable extension of the primer, the run-off product is detected after prolonged incubation (120 min instead of 30 min)—an activity of pRN1 primase which we refer to as “DNA polymerase mode.”

Thus, the “primase mode” is only activated when the GTG motif is present in the template and the primer contains the genuine 5′ end structure bearing a 5′ triphosphorylated ribonucleotide. Only under these restrictive conditions is it possible to restart primer synthesis from a preformed primer/template duplex. We conclude that the primase activity depends upon contacts with the 5′ end of the primer and the GTG motif in the template strand during the primer synthesis reaction. This is in line with our structural investigations which demonstrated that the GTG motif and nucleoside triphosphates are bound by the HBD (Boudet et al., 2019).

Templates devoid of the GTG motif are only weakly bound by the HBD. Similarly, a 5′ primer end lacking triphosphorylation is likely to be bound more weakly by the HBD since it is known that HBD harbors two nucleotide binding sites that bind the nucleotides mainly *via* the triphosphate groups. Consequently, activation of the “primase mode” can only occur if the 5′ primer end hybridized to the GTG containing template is bound and orientated by the HBD. In the absence of this specific activation, the primase remains in “DNA polymerase mode,” resulting in a much slower elongation reaction unable to terminate at a specific unit length. We consider this polymerase activity not physiologically significant if compared with the much more processive cellular DNA polymerases. Absence of proofreading and processivity in the polymerase mode of pRN1 primase suggest this enzyme provides replication initiation at the recognition sequence but not the main polymerase function which is likely provided by cellular enzymes.

Next, we compared the kinetics of nucleotide incorporation by pRN1 primase in “primase” and in “polymerase” mode. To this end, we quantified the appearance of the respective reaction products in the primase assay *via* HPLC analysis (Table 2). We found that the DNA polymerase activity of pRN1 primase occurs with a relatively low rate (∼0.1 mol dNTP per mol enzyme incorporated per minute), however, when a GTG containing template or a genuine primer/template is offered (“primase mode”), the incorporation rate increases about 20-fold (see Table 2).

Mechanistically this activation is likely caused by binding and orientation of the DNA substrate by the HBD in preparation for the enzymatic reaction occurring at the active center of the catalytic domain as discussed above. We therefore predict that the HBD is not only required to initiate dinucleotide synthesis but that the HBD remains bound to the primer/template throughout the complete primase extension cycle until an eight nucleotide unit length primer has been generated. This raises the question whether the HBD is also involved in the termination of primer synthesis occurring as soon as a unit length has been reached by the catalytic domain. The experimental data presented in Table 2 further show that dNTP incorporation in polymerase mode is slightly more rapid with the construct lacking the C-terminal domain. This observation may be interpreted as a hindrance of the polymerase activity harbored in the N-terminal domain if the HBD binds primer-templates that lack the triphosphate or the recognition sequence and hence do not lead to activation of “primase mode.”

### The Interdomain Linker Length Influences Primase Activity and Primase Abortion Rate

**FIGURE 3:**
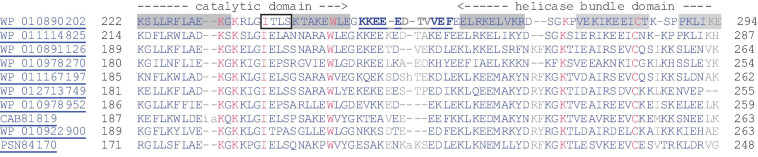
Alignment of the region between the catalytic domain and the HBD of the pRN1 replication enzyme with homologous archaeal replication proteins, including those encoded by mobile genetic elements (pRN1, pRN2, pMEN7, and pDL10). The sequence of the pRN1 replication protein is given in the first line with helices highlighted with gray background and the beta-strand boxed. The residues in the linker sequence which could not be resolved in the crystal structure (pdb: 31M1) are underlined and bold. The alignment was generated with COBALT at a 4 bit threshold for coloring.

The pRN1 primase is a single subunit archaeo-eukaryotic primase offering the unique possibility to study the interaction between the catalytic domain and the HBD in more detail by mutating the linker sequence between both domains. The flexible linker sequence (G249–F260) between the domains is flanked by α-helical secondary structure elements of the catalytic domain and the HBD. Phylogenetically the linker sequence itself is partially conserved (refer to sequence alignment in Figure 3) containing a high fraction of charged amino acids. The linker length varies by up to two residues longer or up to two residues shorter as observed in the close homologs of pRN1 primase.

Since the linker sequence is partially conserved we first tested the effect of substituting the linker sequence by an increasing number of glycine-serine repeats. With this approach, we sought to avoid possible complications arising from residues potentially exerting a specific effect on the primase activity. The mutants with the increasing number of serine-glycine repeats were expressed recombinantly in *E. coli* and the purified protein was assayed using an HPLC-based primase activity assay. In this assay a short oligonucleotide with the primase recognition motif 5′-GTG-3′ is supplied as a template and in the presence of dATP and rATP on average eight (seven to ten) nucleotide long primer solely consisting of adenine bases is synthesized. Amounts of the different primase products were quantified by integrating the corresponding peak areas. We distinguish between the aborted primers (the dinucleotide pppaA as well as the trinucleotide pppaAA; lower case letters ribonucleotide, upper case letters deoxynucleotide), intermediate products with lengths between four and six nucleotides and unit-length primers of 7–10 nucleotides. With the wild-type enzyme the observed products are mainly unit-length primers and aborted products and almost no intermediate products (see Supplementary Figure 1).

The dinucleotide is released if the first phosphodiester bond forming step (the condensation of the initiating rATP with the first elongating dATP) is not followed by further elongation of the dinucleotide/template. Since the dinucleotide/template hybrid is unstable, lack of productive elongation might result whenever repositioning of the dinucleotide/template at the active site of the catalytic domain occurs too slowly, leading to release of the dinucleotide from the enzyme-substrate complex. The same reason might explain why a small amount of the trinucleotide pppaAA is also released as observed in the assay. Further intermediate primer products are barely observed, pointing to a more efficient repositioning as soon as the primer reaches a length of four nucleotides.

**FIGURE 4:**
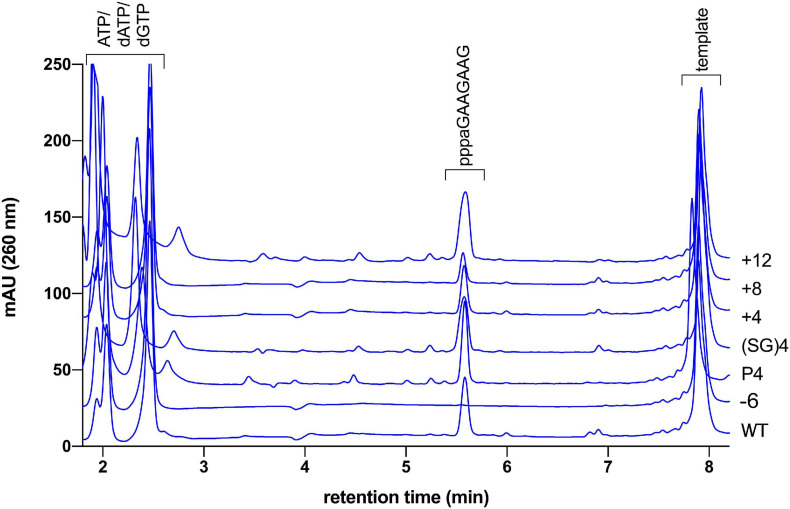
HPLC based primer length determination of linker length mutants. Primase assay of wild type primase incubated with template “mixed” produces a unit length primer of 8 nt (ppppaGAAGAAG). The same main product is observed in varying amount with the linker mutants Δ6, P4, (SG)_4_, +4, +8, and +12. The identity of the product was confirmed to be the ppppaGAAGAAG with MALDI-TOF mass spectrometry (calculated exact mass 2,746.38 gmol^–1^, found: 2,746.04).

In our standard assay with the substrate T_8_GTGT_3_ we would expect to observe only primers with the unit-length of eight nucleotides. However, products of the length of seven, nine, and 10 nucleotides are also observed. The same length distribution is observed if the longer template T_14_GTGT_3_ is used instead. We explain the occurrence of these longer products as well as the seven nucleotide long primer with a slippage of the primer on the homonucleotide stretch of the template during the primase elongation cycles. This explanation is supported by the observation that a template with a mixed base sequence downstream of the GTG motif leads to the exclusive formation of the 8 nt long primer (see also Figure 4).

All mutants with the increasing number of serine-glycine repeats show greater activity than the wild-type protein (Table 3). Thus, it appears that a more flexible linker favors primer synthesis. However, the increase of activity is limited to twofold of the wild-type activity, which is already reached if one serine-glycine repeat is incorporated in the linker sequence. A further increase in backbone flexibility does not increase the enzymatic activity further. To substantiate the relationship between backbone flexibility and activity we next substituted the glycine residues of the (SG)_4_ mutant with prolines in order to obtain linkers with decreased flexibility. As can be seen in Table 3 the decrease of linker flexibility by an increasing numbers of incorporated proline residues is well correlated with a decrease in primase activity. We also note that the fraction of aborted primers is negatively correlated with the enzymatic reaction rate.

**TABLE 3:** Primase activity of mutants with differing linker backbone flexibility (template T_8_GTGT_3_).

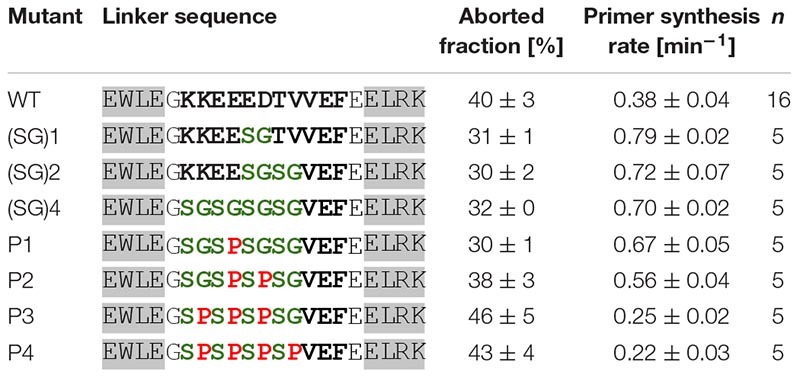

*For each mutant the fraction of aborted primers (dinucleotide and trinucleotide) and the primer synthesis rate is given. The primer synthesis rate relates to the amount of synthesized primer between seven and 10 nucleotides. The number of replicate measurements is given in column *n*. The letters in bold indicate the peptide stretch not resolved in X-ray structure determination.*

Next, we investigated the effect of linker length on enzymatic activity. For this purpose, mutants with different numbers of serine-glycine repeats were produced and tested (Table 4). The highest enzymatic activity was observed with the mutant having four additional amino acids in the linker (two additional serine-glycine repeats). Further extension of the linker decreases primase activity. We attribute this to the reduced local concentration of the HBD relative to the active site. The observed tendency is in agreement with the fact that a mixture of the separate domains does not display any primase activity.

**TABLE 4:** Primase activity of linker length mutants (template T_8_GTGT_3_).

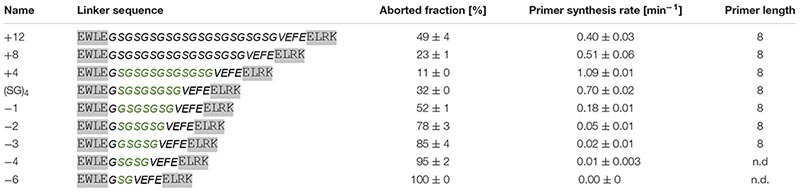

*For each enzyme the fraction of aborted primers (dinucleotide and trinucleotide) and the primer synthesis rate was measured (*n* = 5). In addition the main primer product length with the mixed sequence template is given.*

Upon shortening the linker, the enzymatic activity is strongly reduced. Truncating the linker by only one amino acid already reduced the activity about threefold. The even shorter variants display a progressively greater loss of activity. Noteworthy, as with the linker flexibility series (Table 3) we find that the fraction of aborted primers is negatively correlated with the enzymatic reaction rates of the linker length mutants.

### The Unit-Length of the pRN1 Primase Primer Is Eight Nucleotides Regardless of Interdomain Linker Length

The mechanism of primer termination of pRN1 primase is not well understood. Our data suggest that in the “primase mode” the primer/template end remains attached to the HBD. Therefore possibly, there should be an upper limit on primer length imposed by the length of the linker between the two domains. The “caliper model,” as suggested for *S. solfataricus* primase (Yan et al., 2018), would predict the unit-length of the primer to be determined by the maximal attainable distance between the primer 5′ end binding site on the HBD and the active site of the catalytic domain (where the 3′-end must be bound).

To test whether this mechanism of primer termination also applies to pRN1 primase we determined the primer lengths produced by our linker length mutants. As the ssDNA template we used an oligonucleotide with a mixed nucleotide composition upstream of the 5′-GTG-3′ motif. The mixed sequence is intended to prevent slippage of the primer along the template, and indeed we only obtained a single product of eight nucleotides in this variant of the assay (Figure 4). Surprisingly, the linker mutants varying in linker length by 15 amino acid residues (mutant +12 vs. mutant −3) all produce a linker of eight nucleotides. We can therefore exclude the possibility that primer termination proceeds according to the “caliper model.”

### How Is Primer Termination Triggered?

The ability to terminate elongation after reaching unit length may be considered a defining property of the primase mechanism as opposed to processive polymerases since it limits the amount of nucleotides incorporated by the error-prone primase to a minimum before a high-fidelity DNA polymerase takes over, thereby minimizing the amount of proofreading required to process Okazaki fragments and speeding the replication process. Our data clearly shows that primer termination is not influenced by the linker length—thus we exclude the “caliper” model.

Our experiments do not support primer length determination by stochastic dissociation from the substrate primer-template either. Firstly, with a template devoid of homonucleotide runs, we strictly observe only primers with a length of 8 nt, whereas spontaneous dissociation would by necessity result in a wider distribution of primer lengths. Secondly, if a preformed primer-template duplex is incubated with primase in the presence of the cognate dNTPs, the stochastic model would predict elongation by the same number of nucleotides as are used in a unit length primer. This is, however, not the case, since elongation occurs only up to the overall unit-length.

**FIGURE 5:**
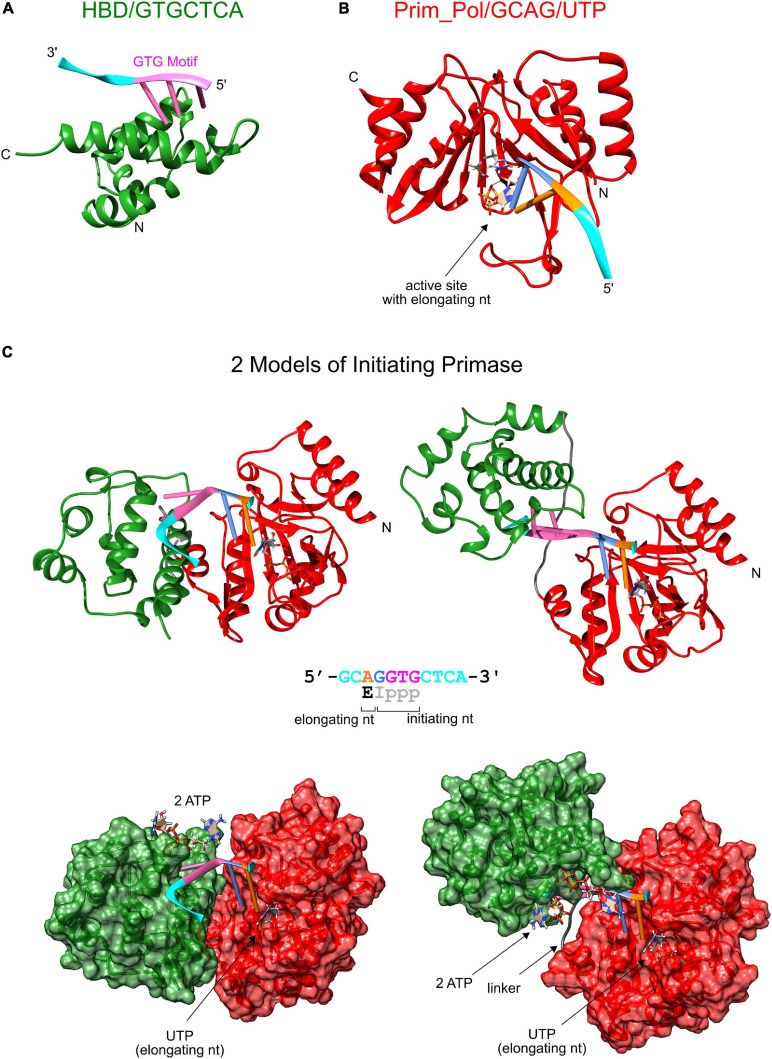
Structure model of the initiating primase. **(A)** Solution structure of the HBD domain (green) binding sequence-specifically a template with a GTG motif (pdb: 6GVT), **(B)** Model of catalytic Prim_Pol domain (red, pdb: 3M1M) complexed with template and UTP at the elongating position (derived from M. tuberculosis PolDom (pdb: 4MKY and 3PKY) having the same conserved active site). **(C)** Two models of the complete primase during initiation of primer synthesis. The two models were obtained by rigid body docking between the protein complexes of Prim_Pol/GCAG and HBD/GTGCTCA while enforcing a covalent bond between the two terminal guanosines and modeling a flexible linker between both domains. The dinucleotide is formed opposite of the two bases upstream of the GTG motif and constrains the possible conformations of the initiating primase as the GTG remains bound to the HBD domain and dinucleotide formation is catalyzed at the active site of the Prim_Pol domain.

The third proposed model of primer termination explains the specific termination at unit length primers through a steric clash resulting from the rotation of the domains which is induced by the helical conformation of the primer-template. This model is in principle compatible with our observations since the clash and ensuing primer termination might occur at a specific angle dictated by the geometry of the protein structures of the two domains and not by the linker length. To gain better structural understanding whether such a termination mechanism is plausible, we modeled the structure of the active primase-substrate complex. Starting point for this model was the NMR structure of the HBD sequence specifically binding the GTG motif in the template ssDNA strand [pdb: 6GVT, (Boudet et al., 2019)]. The initial dinucleotide of primer synthesis is formed complementary to the two bases directly 5′ of the GTG motif. Therefore these two bases of the template must interact with the active site of the Prim_Pol domain. Currently there is no direct structural information of the liganding of template and nucleotides in the active site of pRN1 primase. However the active site of pRN1 primase shares structural homology with the Polymerase Domain (PolDom) from *Mycobacterium tuberculosis* Ligase D, an enzyme involved in non-homologous end joining. Two crystal structures of this enzyme liganded with template ssDNA and a UTP molecule at the elongating position have been determined [pdb: 3PKY (Brissett et al., 2011) and 4MKY (Brissett et al., 2013), respectively]. We used this information to place UTP and a four base template strand in the crystal structure of the pRN1 primase [pdb: 3M1M, (Beck et al., 2010)]. Next, the two separate domains (Figures 5A,B) underwent a rigid body docking while enforcing the covalent linkage of two the domains *via* the flexible linker (wild type sequence) and fusing the two template parts into one continuous DNA template. During the docking procedure the two template parts were restrained relative to the interacting protein surfaces and the covalent bonds at the fusion site in the single-stranded DNA backbone were sampled from an RNA rotamer library. With this procedure we arrived at two biochemically and structurally plausible models (Figure 5C). The conformations differ considerably between the two models: In one model (left) the template is bent at the fusion site between the GTG motif (magenta) and the base opposite to the initiating base (blue). In this model the connecting linker (gray) is not fully extended which is in line with our experimental observation that even shortened linkers support primer synthesis (see Table 4). The alternative model (Figure 5C, right) has a nearly linear (but twisted) DNA backbone conformation. In this case the linker is stretched to connect both domains. This structure could more easily explain the recognition of the terminal triphosphate group of the primer by the HBD, which we biochemically observed to be essential for primer synthesis together with the ribose at the 5′ primer end. In the NMR structure of the quaternary complex (Boudet et al., 2019), HBD binds both ATPs *via* the triphosphates. One triphosphate moiety is in close proximity to the template backbone and the adenine base is close to the template base opposite of the initiating nucleotide (blue). Thus it is possible that the triphosphate of the 5′ primer end (which is part of the initiating nucleotide) could displace the triphosphates of one of the ATPs bound by HBD during primer synthesis initiation.

In summary, the well-regulated primer termination was found to be dependent on interaction with the 5′ terminus, yet it is not the result of the flexible interdomain region acting as a molecular caliper to restrict the distance that can be accommodated between the binding sites in the two domains.

The rotational clash model advanced to explain this regulation in eukaryotic primases (Baranovskiy et al., 2016b) suggests primer termination would occur by the relative motion of the domains dictated by the growing double-stranded helical DNA between the Prim_Pol and the HBD. In our models the rotation and translation of the HBD relative to the Prim_Pol domain would first shorten the distance covered by the flexible linker. Thus primer synthesis beyond the dinucleotide would be accommodated. When the primer length approaches eight nucleotides (i.e., ∼24 Å of double-stranded DNA) both domains will be quite distant from each other which renders a rotational clash between both domains doubtful.

It is difficult to fully explain the observed stringent primer termination by the currently proposed mechanisms of primer termination. We found experimentally that the HBD is crucial for primer synthesis and that the covalent linkage between both domains is required for efficient primer synthesis. We argue that the main function of the HBD is to prepare primer synthesis by binding and orientating the substrates. In fact without the HBD, primer initiation is not possible and elongating dNTP incorporation is slowed by about a factor of 20. On this basis we suggest that efficient activity in “primase mode” is stimulated by contacts between the two domains, which thus act cooperatively in this enzyme-subtrate complex. Such specific interaction would depend upon correct arrangement of the domains relative to each other and the nucleic acid substrates, which appears to be determined by the presence of the interdomain linker and the binding of both primer ends as supported by our experiments. Upon exceeding unit length the complex would reach a conformation that no longer permits the activity promoting interaction of the domains either as a consequence of increasing flexibility or due to the increased length of dsDNA separating them.

Our investigations of the primase extension cycle demonstrated that the substrate requirements for genuine primase activity can be explained if the triphosphorylated 5′ primer end together with the motif GTG remains bound to the helix bundle domain HBD. Variation of the flexibility and the length of the interdomain linker between catalytic domain and HBD showed a profound effect on primase activity and primer abortion rate but primer termination continued to occur robustly at eight nucleotides. Capturing the structures of the enzyme-substrate complex during the elongation cycle will be required to decide how termination is triggered by the lack of stimulation of the HBD enforced by the growing primer/template.

## Data Availability Statement

The original contributions presented in the study are included in the article/Supplementary Material, further inquiries can be directed to the corresponding author/s.

## Author Contributions

JB performed the experiments reported in Figures 2, 4, Table 2, and Supplementary Figures. JB and J-CD wrote the manuscript. J-CD performed the experiments reported in Tables 3, 4. GJ performed the model calculation reported in Figure 5. GL directed the project, obtained funds and wrote the manuscript. All authors contributed to the article and approved the submitted version.

## Conflict of Interest

The authors declare that the research was conducted in the absence of any commercial or financial relationships that could be construed as a potential conflict of interest.
