# Supplementary material for: Stringent Primer Termination by an Archaeo-Eukaryotic DNA Primase
Source: Front Microbiol. 2021 Apr 13;12:652928. doi: 10.3389/fmicb.2021.652928 (PMC8076596; doi:10.3389/fmicb.2021.652928)
Supplement: Supplementary file 1 [file Data_Sheet_1.PDF]

Stringent Primer Termination by an Archaeo-Eukaryotic DNA Primase

Supplementary Information

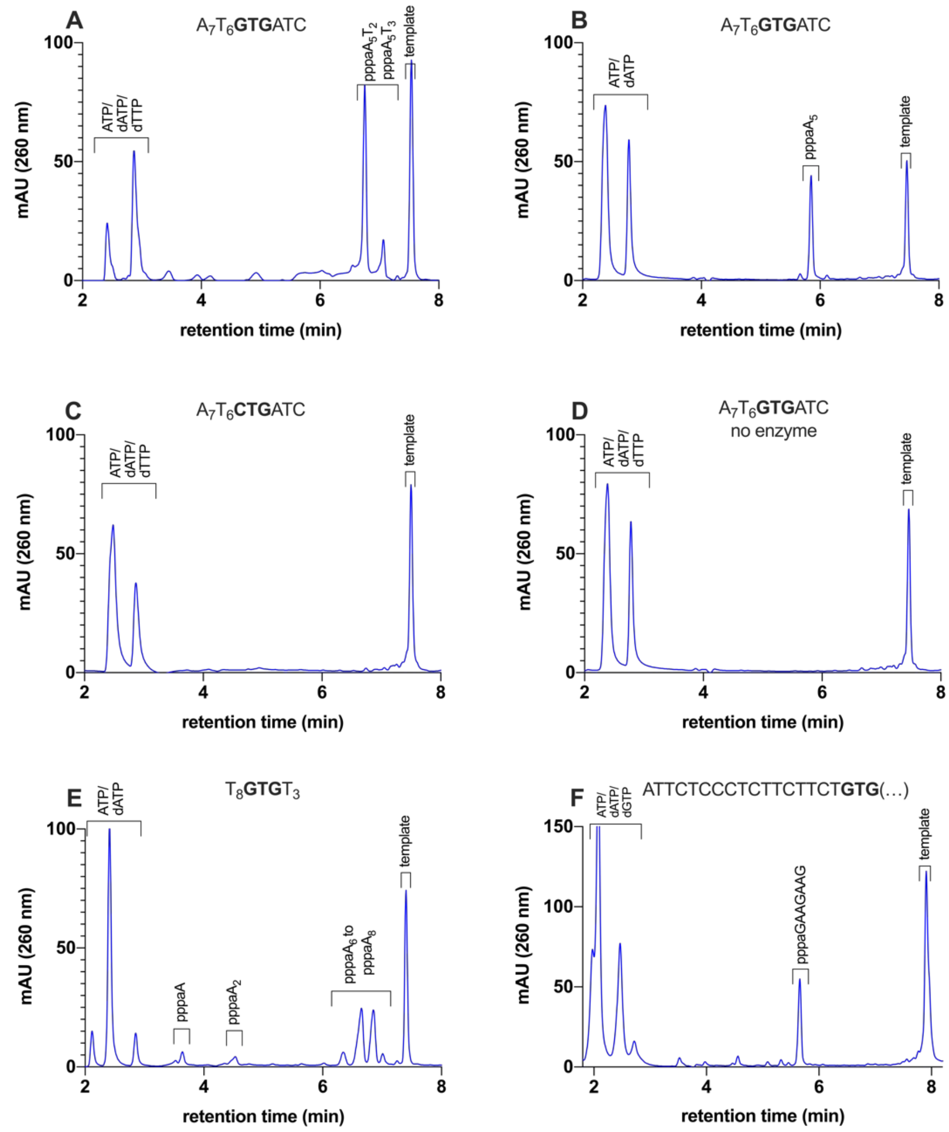

Supplementary Figure 1: *HPLC analysis of the primase reactions.*

A *Primase assay with template ssDNA "GTG" (A_7T_6**GTG**ATC) in presence of ATP, dATP and dTTP. The main products are the 8 nt and 9 nt primers pppaA_5T_2 and pppaA_5T_3.* **B** *Assay with template ssDNA "GTG" (A_7T_6GTGATC) in presence of ATP and dATP. The main product is the 6 nt primer pppaA_5 (6 nt primer with triphosphorylated ribonucleoside at 5' terminus). Identity of peaks was verified by preparative HPLC and MALDI-TOF mass spectrometry (Supplementary*

Figure 2). **C** *No primers are formed with a template devoid of the motif GTG (A_7T_6**CTG**ATC) in presence of ATP, dATP and dTTP.* **D** *Negative control without enzyme: no primers are observed, conditions otherwise identical to* ***A***. **E** *Assay with template ssDNA T_8**GTG**T_3 yields primarily unit length primers with 7, 8 or 9 nt length ($pppaA_6$ to $pppaA_8$). Moreover small amounts of the abortive primers pppaA and $pppaA_2$ are formed. All reactions were set up as described in methods and materials.* **F** *A single product with a length of 8 nt (pppAGAAGAAG) is formed on a mixed-sequence template, in contrast to the slippage occurring on homopolymeric template sequences.*

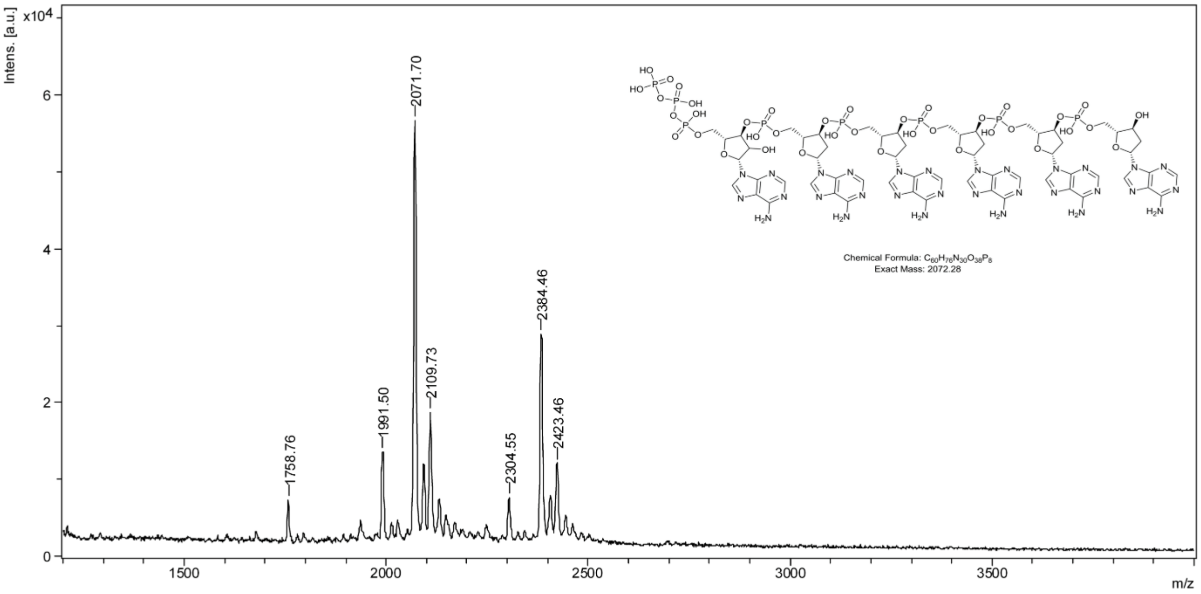

Supplementary Figure 2: *MALDI-TOF mass spectrum of the substrate $pppaA_5$.*
Calculated exact mass 2072.28 $gmol^{-1}$, found: 2071.70 (M-H). The molecular mass of 2384.46 corresponds to the product $pppaA_6$.

Primer	ε [L mol^{-1} cm^{-1}]	mol product per mol enzyme / peak area [mAU^{-1} min^{-1}]
**aA5**	91199	4.386
**aA6**	106405	3.7592
**aA7**	121595	3.2896
**aA8**	136799	2.9239
**aA9**	151999	2.6316
**aGAAGAAG**	112032	3.5704
**aA5T7 (full length)**	159600	2.5063
**aA5T**	102000	3.9216
**aA5T2**	111600	3.5842
**aA5T3**	121200	3.3003

Supplementary Table 1: *Conversion factors for HPLC based quantification for reactions under standard conditions, i.e. 0.25 μM enzyme and injection volume of 20 μl. The extinction coefficients are calculated with Scipps Research Institution Calculator (https://www.scripps.edu/researchservices/old/corefac/biopolymercalc2.html).*

Calculation of dNTP incorporation rates given in table 2 from HPLC data

Analyte	ret time [min]	integral [mAU*min]	mol product/min/mol enzyme	std dev	dNTP incorporated/min/molecule enzyme	std dev dNTP/min
**A6_GTG + primase**						
exp1 (full length)	7.2903	0.4838	1.2126			
exp2 (full length)	7.2931	0.5205	1.3046			
exp3 (full length)	7.2980	0.5464	1.3694			
exp4 (full length)	7.2831	0.5224	1.3093			
exp5 (full length)	7.2909	0.5351	1.3410			
average			1.3074	0.0591	0.0763	0.0034
**A6_GTG + PPD**						
exp1 (full length)	7.2879	0.6478	1.6236			
exp2 (full length)	7.2921	0.8140	2.0402			
exp3 (full length)	7.2959	0.7270	1.8221			
exp4 (full length)	7.2950	0.7586	1.9012			
exp5 (full length)	7.2879	0.7052	1.7675			
average			1.8309	0.1548	0.1068	0.0090
**A6_CTG + primase**						
exp1 (full length)	7.2881	0.4083	1.0233			
exp2 (full length)	7.2924	0.4170	1.0452			
exp3 (full length)	7.2938	0.2986	0.7484			
exp4 (full length)	7.3024	0.2665	0.6679			
exp5 (full length)	7.3008	0.2766	0.6932			
average			0.8356	0.1838	0.0487	0.0107
**A6_CTG + PPD**						
exp1 (full length)	7.3061	1.4864	3.7254			
exp2 (full length)	7.3059	1.8315	4.5903			
exp3 (full length)	7.3055	1.5251	3.8225			
exp4 (full length)	7.3114	1.6342	4.0958			
exp5 (full length)	7.3037	1.1579	2.9021			
average			3.8272	0.6166	0.2233	0.0360

**A6_GTG + primase**						
**Analyte**	**ret time [min]**	**integral [mAU*min]**	**mol product/min/mol enzyme**	**std dev**	**dNTP incorporated/min/molecule enzyme**	**std dev dNTP/min**
exp1 (7nt)	7.1235	0.8822	3.4597			
exp2 (7nt)	7.1216	0.5626	2.2063			
exp3 (7nt)	7.1161	0.6176	2.4219			
exp4 (7nt)	7.1163	0.6091	2.3888			
exp5 (7nt)	7.1239	0.5671	2.2240			

average			2.5401	0.5230	0.1270	0.0523
exp1 (8nt)	7.2772	2.0587	7.3788			
exp2 (8nt)	7.2756	2.4168	8.6622			
exp3 (8nt)	7.2799	2.0873	7.4815			
exp4 (8nt)	7.2771	2.0440	7.3260			
exp5 (8nt)	7.2726	1.8965	6.7973			
average			7.5292	0.6866	1.1294	0.0687
exp1 (9nt)	7.3968	0.3781	1.2478			
exp2 (9nt)	7.3970	0.1463	0.4830			
exp3 (9nt)	7.4136	0.1440	0.4753			
exp4 (9nt)	7.3996	0.0233	0.0770			
exp5 (9nt)	7.3862	0.1596	0.5268			
average			0.5620	0.4244	0.0843	0.0424
exp1 (sum 7 – 9 nt)		3.3190	12.0864			
exp2 (sum 7 – 9 nt)		3.1257	11.3515			
exp3 (sum 7 – 9 nt)		2.8489	10.3787			
exp4 (sum 7 – 9 nt)		2.6764	9.7918			
exp5 (sum 7 – 9 nt)		2.6232	9.5480			
average			10.6313	1.0698	1.3407	0.1070

**GTG + primase**						
**Analyte**	**ret time [min]**	**integral [mAU*min]**	**mol product/min/mol enzyme**	**std dev**	**dNTP incorporated/min/molecule enzyme**	**std dev dNTP/min**
exp1 (7nt)	6.7328	0.6651	2.5002			
exp2 (7nt)	6.7434	0.7049	2.6499			
exp3 (7nt)	6.7439	0.7055	2.6522			
exp4 (7nt)	6.7537	0.7715	2.9001			
exp5 (7nt)	6.7548	0.7005	2.6333			
average			2.6671	0.1448	0.9335	0.1013
exp1 (8nt)	6.9542	1.0978	3.6113			
exp2 (8nt)	6.9563	1.1472	3.7737			
exp3 (8nt)	6.9432	1.1960	3.9342			
exp4 (8nt)	6.9674	1.1379	3.7432			
exp5 (8nt)	6.9669	1.1802	3.8822			
average			3.7889	0.1262	1.5156	0.0505
exp1 (9nt)	7.0976	0.3583	1.0477			
exp2 (9nt)	7.0989	0.2433	0.7112			

exp3 (9nt)	7.0828	0.3690	1.0790			
exp4 (9nt)	7.1133	0.2471	0.7226			
exp5 (9nt)	7.1170	0.3049	0.8916			
average			0.8904	0.1736	0.4007	0.1215
exp1 (sum 7 – 9 nt)		2.1212	7.1592			
exp2 (sum 7 – 9 nt)		2.0953	7.1348			
exp3 (sum 7 – 9 nt)		2.2705	7.6654			
exp4 (sum 7 – 9 nt)		2.1565	7.3659			
exp5 (sum 7 – 9 nt)		2.1856	7.4071			
average			7.3465	0.2154	2.8498	0.0862
